# Tablet-Based Neurocognitive Tests for Ugandan Adolescents Living With HIV: Cross-Sectional Validation Study

**DOI:** 10.2196/103288

**Published:** 2026-10-13

**Authors:** Reuben N Robbins, Angel Nanteza, Joy Louise Gumikiriza-Onoria, Anthony Santoro, Christopher M Ferraris, Christine Karungi, Daphne Tsapalas, Nana Asiedu, Courtney Kirsch, Mei Tan, Jun Liu, Itziar Familiar-Lopez, Victor Musiime, Sahera Dirajlal-Fargo

**Affiliations:** 1 Department of Psychiatry, Vagelos College of Physicians and Surgeons, Columbia University, 1051 Riverside Dr, New York, NY, 10032, United States, 1 646-774-6968; 2 New York State Psychiatric Institute, New York, NY, United States; 3 Uganda-CWRU Research Collaboration, Kampala, Uganda; 4 Butabika Hospital, Kampala, Central Region, Uganda; 5 Department of Psychiatry, College of Health Sciences, Makerere University, Kampala, Central Region, Uganda; 6 School of Social Policy & Practice, University of Pennsylvania, Philadelphia, PA, United States; 7 Joint Clinical Research Centre, Kampala, Central Region, Uganda; 8 University of California, Irvine, Irvine, CA, United States; 9 Department of Psychiatry, Michigan State University, Lansing, MI, United States; 10 Department of Paediatrics, College of Health Sciences, Makerere University, Kampala, Central Region, Uganda; 11 Feinberg School of Medicine, Northwestern University, Chicago, Illinois, Philippines; 12 Lurie Children's Hospital, Chicago, IL, United States

**Keywords:** HIV infection, neuropsychological tests, adolescent, Uganda, mobile applications, neurocognitive disorders, mobile health, mHealth, validity, *NeuroScreen*, psychometrics

## Abstract

**Background:**

Adolescents living with perinatally acquired HIV (PHIV) remain at risk of neurocognitive difficulties, yet access to valid neurocognitive tests is limited in sub-Saharan Africa (SSA), where most of this population resides. Tests that can be administered by nonspecialist personnel are culturally and linguistically appropriate for SSA’s diverse populations and are brief enough for routine use are needed to expand access, but few such tools exist.

**Objective:**

We adapted *NeuroScreen*, a brief tablet-based neurocognitive test battery, into Luganda and evaluated its validity and acceptability for use among Ugandan adolescents with PHIV.

**Methods:**

In this cross-sectional validation study, 100 Ugandan adolescents aged 11 to 20 years (50 with PHIV; 50 HIV-negative controls) completed the Luganda- or English-language *NeuroScreen* battery and a commonly used traditional test battery. Participants were recruited from an existing longitudinal cardiovascular cohort at the Joint Clinical Research Centre in Kampala, Uganda. We examined convergent validity with correlations between the 2 batteries’ individual and global *z*-scores, known-groups validity with unadjusted and age-adjusted group comparisons, criterion validity by examining classification agreement between the 2 batteries quantified with Cohen κ, and acceptability through self-reported technology use and ease of use.

**Results:**

*NeuroScreen* and the traditional battery global *z*-scores were strongly correlated (*r*=0.75; *P*<.001). Correlations between tests assessing similar domains were generally small to moderate, and were strongest for processing speed, attention, and working memory. Adolescents with PHIV scored significantly lower than controls on global scores for both *NeuroScreen* (*P*=.008) and the traditional battery (*P*=.003), even after adjusting for age. Agreement between batteries in classifying participants ≤1 SD (κ=0.56) and ≤2 SD (κ=0.66) below the global *z*-score mean was moderate, with both batteries classifying more adolescents with PHIV than controls below these thresholds. Although fewer than half of participants (42/97, 43%) had previously used a tablet, most rated *NeuroScreen* as easy to use, and prior tablet experience was not independently associated with *NeuroScreen* performance after age adjustment.

**Conclusions:**

The Luganda-adapted *NeuroScreen* demonstrated convergent, known-groups, and criterion validity in Ugandan adolescents with PHIV. By delivering automated administration, automated scoring, and standardized multimedia instructions in the local language on a portable tablet, *NeuroScreen* addresses several persistent barriers to neurocognitive assessment in low-resource settings, including shortages of specialist personnel, the time burden of traditional batteries, and the scarcity of culturally and linguistically appropriate tools. The *NeuroScreen* battery was found to be highly acceptable among adolescents with little or no prior tablet experience. These findings add to a growing body of evidence supporting tablet-based assessment as a practical and scalable mobile health approach to expanding access to neurocognitive testing. Future research should evaluate test-retest reliability, sensitivity to change over time, normative benchmarks, and implementation within real-world clinical workflows.

## Introduction

Globally, there are approximately 1.57 million (1,200,000-1,900,000) adolescents (10-19 y) living with HIV, most with perinatally acquired HIV (PHIV) and living in low- and middle-income countries (LMICs), with approximately 85% living in sub-Saharan Africa (SSA) [1,2]. Greater access to more effective antiretroviral therapy (ART) has greatly improved their health outcomes [3,4], allowing adolescents with HIV to live well into adulthood [5,6]. Despite this public health success, adolescents with PHIV remain at high risk for a variety of complications, including neurocognitive problems [7,8].

As many as 46% of adolescents with PHIV experience neurocognitive problems [9], despite viral suppression [10,11]. Although both the persistence of HIV and its downstream effects in the central nervous system are thought to play a key role in these complications [12], low socioeconomic status, limited educational opportunities, early life adversity, and other comorbidities are also likely contributing factors [11,13,14]. PHIV typically affects the neurocognitive domains of processing speed and executive functioning [9]. Neurocognitive problems have been associated with poor academic performance, suboptimal ART adherence, increased mental health problems, poor decision-making, and engagement in HIV transmission and other risk behaviors (eg, unprotected sex) [11,15-29].

Early detection of neurocognitive problems can allow for ART regimen adjustments, tailored support approaches, better coping, and earlier intervention [30]. Without timely intervention, these problems may accumulate, limiting adolescents’ future independence, employment potential, and quality of life [31]. However, detection of neurocognitive problems is challenging in LMICs for several reasons [32,33]. First, many LMICs, including those in SSA, have a limited number of psychologists and/or neuropsychologists trained to administer, score, and interpret traditional neuropsychological test batteries to measure and diagnose neurocognitive problems [34]. For example, in Uganda, where the current study took place, there are only 0.04 psychologists per 100,000 people, compared with 31.3 per 100,000 in the United States [35,36]. The precise number of neuropsychologists and/or psychologists with specialized neuropsychology training in Uganda is not known.

Second, access to culturally and linguistically appropriate neurocognitive tests is limited. Most tests to date were designed for use by English speakers in high-income countries and, as such, may only be available in English, which may be inappropriate for other cultures and contexts [37-39]. While English is often considered an official language of government and taught in schools in many LMICs, including Uganda, many communities rely on their indigenous languages and local dialects for everyday activities and may not be fully fluent in the officially recognized languages [37,40]. Administering English-language tests in communities that prefer or rely on other languages not only produces invalid test results but also violates ethical practices for psychologists and standardized testing practices [41,42]. Finally, traditional test batteries often require multiple hours to complete—time and resources that overburdened and resource-constrained health care systems in LMICs do not have.

Computerized neurocognitive tests, particularly those that are tablet-based, show promise for use in LMICs and may help make testing more accessible to LMIC populations [43-45]. Compared with traditional tests, computerized tests are highly automated, and many have been designed for administration by a wide variety of personnel who do not have specialized training. Tablet-based tests are also highly portable, and tablets are considerably more affordable than desktop or laptop computers [46,47].

*NeuroScreen* is a tablet-based battery of neurocognitive tests designed for resource-limited settings [48-50]. It has been validated for use in adult populations with HIV in South Africa [48] and among patients with first-episode psychosis in Uganda [51], as well as with adolescents and young adults with PHIV in Thailand [49] and the United States [39]. This cross-sectional validation study evaluated the construct validity (ie, convergent validity, known-groups validity, and criterion validity), as well as acceptability, of a Luganda-language–adapted *NeuroScreen* for use among adolescents in Uganda living with PHIV. We hypothesized that performance on the *NeuroScreen* battery would be positively correlated with performance on a traditional battery of neurocognitive tests assessing similar domains, that adolescents with PHIV would perform worse than controls on both batteries, and that the 2 batteries would show agreement in the proportion of participants scoring ≤1 SD and ≤2 SD below the global mean.

## Methods

### *NeuroScreen* Translation Process From English to Luganda

Prior to data collection, adaptation of the English *NeuroScreen* test instructions and content into Luganda was completed. We followed a “group consensus” [52] process developed in our laboratory and informed by the International Test Commission [53,54], the American Educational Research Association [41], Nguyen et al [55], and Brislin [56], along with our research team’s experience adapting neurocognitive and other measures cross-culturally. Our process began with generating a set of forward translations (English to Luganda) by a professional translator. Next, 4 bilingual (English and Luganda) individuals (1 Master’s-level psychologist, 2 research psychometrists, and 1 research assistant) with experience administering traditional neurocognitive tests in Uganda completed blinded, individual back-translations. Back-translators were asked to make notes and identify any Luganda translations that were not easily understood. Then the US-based team reviewed the back-translations to identify differences that could affect the meaning and understandability of the instructions (ie, consistency with the original English and across the back-translations), including any translation issues identified by the translators. A series of 4 meetings with the United States and Ugandan teams was convened to review all translations, with special attention paid to those with identified issues. Final Luganda translations were generated after consensus of the translators was achieved. Some examples of issues addressed were overly technical words and English words without an equivalent in Luganda. For example, the Luganda word for circle is “obukulungwa” but was not readily known by any of the bilingual translators. During the group consensus, the bilingual translators reported that they could infer the word from the context in which it was used (instruction sets for the Trail Making tests) but had not encountered it before. Two of the bilingual translators reported looking up the word in a Luganda dictionary and confirming that it meant circle. Hence, the team decided that an Anglicized version, “esaako,” would be much more widely understood by Luganda speakers. Once translations were finalized, audio recordings were made, synced with video instructions, and added to the *NeuroScreen* app.

### Participants

In this cross-sectional validation study with a known-groups comparison, participants were 100 Ugandan adolescents (11-20 y): 50 living with PHIV and 50 HIV-negative controls recruited from the same communities as those with PHIV. All participants were recruited from a longitudinal study of cardiovascular health in adolescents with PHIV (K23HD088295; PI: SDF). Participants were included if they were (1) 11 to 20 years old, (2) enrolled in the cardiovascular study, and (3) had documented PHIV or HIV-negative status; participants with PHIV were additionally required to (4) have been on an ART regimen for the last 6 months, and (5) be virally suppressed (<200 copies/mL) at the time of enrollment in the parent study. For the primary correlation of interest (global *z*-scores between the 2 batteries), a sample size of 100 provides ≥80% power to detect a correlation of ≥0.28 in absolute value (a small effect size).

### Procedures

This study was carried out at the Joint Clinical Research Centre (JCRC) in Kampala, Uganda, between February 2021 and November 2022. Parents or caregivers of potential participants were approached by study counselors at the JCRC and given information about the study. Trained research assistants collected participants’ sociodemographic information before administering the *NeuroScreen* battery on a Samsung Galaxy Tab A 10.5-inch tablet. Participants were given the option to complete *NeuroScreen* in their language of preference, English or Luganda. After completing *NeuroScreen* and taking a short break, participants completed a traditional battery of pencil-and-paper neurocognitive tests and questionnaires.

### Ethical Considerations

This study was reviewed and approved by the Uganda National Council for Science and Technology (#HS2266), the JCRC Research Ethics Committee (Uganda), the New York State Psychiatric Institute Institutional Review Board (IRB #8029), and the University Hospitals Cleveland Medical Center IRB (#11-16-18). All procedures were conducted in accordance with the ethical standards of these committees and with the 1964 Declaration of Helsinki and its later amendments. This study was reported according to the STROBE (Strengthening the Reporting of Observational Studies in Epidemiology) guidelines. The completed STROBE checklist is provided as Checklist 1.

Written informed consent was obtained from all participants aged 18 years and older. For participants younger than 18 years, written informed consent was obtained from a parent or caregiver, and written assent was obtained from the adolescent. Participants and caregivers were informed that participation was voluntary, that they could decline to answer any question, and that they could withdraw from the study at any time without penalty or loss of benefits to which they were otherwise entitled. All study data were deidentified and stored using unique study identification numbers rather than personal identifiers. Data were kept on password-protected, access-restricted secure servers available only to authorized members of the study team.

Participants were compensated 186,000 UGX (approximately US $50 at the time of the study) for their time and travel. The amount was determined in accordance with local norms and approved by the reviewing ethics committees.

### Measures

#### Sociodemographics

Sociodemographic information from caregivers and participants included participants’ age, sex, years of education, and household income. Current HIV medical data were obtained from participants’ research records and included the most recent viral load and CD4 cell count, as well as the nadir CD4 cell count and years on ART.

#### Technology Use and *NeuroScreen* Acceptability

Experience and comfort using digital technologies (ie, desktops and laptops, smartphones with touchscreens, and tablets) were collected with a 12-item questionnaire that asked participants about their experience with computers, smartphones, and tablets (eg, “Have you ever used a smartphone with a touchscreen before?,” “How often do you use a smartphone with a touchscreen?,” and “How comfortable are you using a smartphone with a touchscreen?”). Responses were either yes/no, frequency-based (eg, every day, 1‐3 times per week, or less than once a month), or on a 6-point Likert-type scale (eg, very comfortable to very uncomfortable).

#### NeuroScreen

*NeuroScreen* was administered on Samsung Galaxy Tab A 10.5-inch tablets running Android version 10 or 11 by either our Ugandan psychologist (JLG-O) or our research assistant (AN). Both received approximately 2 hours of direct training in the administration of *NeuroScreen*, in addition to a review of the user manual and multiple (~10) practice assessments. *NeuroScreen* was administered in a private and quiet office space and took, on average, 25 minutes to complete. The test battery is embedded in a graphical user interface that allows the examiner to enter participant data and administer the tests. To reduce potential bias, the examiner read standardized test instructions presented on the screen for the verbal learning and memory and verbal working memory tests; embedded instructional videos in the local language were played for the remaining tests. The app automatically cycled through the battery of tests in the same sequence and forced delayed recall of the verbal memory test after a 5-minute delay. After completing all subtests, results were automatically uploaded to a secure server. Each test is automatically scored, thus reducing potential bias. Each test yielded a primary score that was used for analysis. The battery (Table 1) included 5 tests of processing speed, 1 test of executive functioning, 1 test of verbal learning and delayed recall, 1 test of motor speed (finger tapping with dominant and nondominant hands), and 1 test of working memory (number span forward and backward) for a total of 12 scores. For details, see Robbins et al [49].

**Table 1.** *NeuroScreen* and traditional test batteries by domain and test.

Domain	*NeuroScreen* battery	Traditional battery
Learning	Verbal list learning (VLL)	KABC-II^a^: Atlantis (ATL) [57] KABC-II: Hand movements (HM) [57]
Memory	Verbal list learning delayed recall (VLLD)	KABC-II: Atlantis delayed (ATLD) [57]
Working memory	Number span forward (NSPNF) Number span backward (NSPNB)	WISC-IV: Digit span forward (DSF) [58] WISC-IV: Digit span backward (DSB) [58]
Executive functioning	Trail Making Test 2 (TM2)	Children’s Color Trails Test 2 (CCTT2) [59] KABC-II: Pattern reasoning (PR) [57] KABC-II: Rover (RV) [57]
Processing speed	Visual discrimination 1 (VD1) Visual discrimination 2 (VD2)	WISC-IV: Symbol search (SS) [58] WISC-IV: Coding (CD) [58]
Motor speed	Trail Making Test 1 (TM1) Number speed test (NSPD) Trail Making Test 3 (TM3) Finger tapping dominant hand (FTD) Finger tapping nondominant hand (FTND)	CCTT1 [59]

a KABC-II: Kaufman Assessment Battery for Children, Second Edition.

#### Traditional Test Battery

Participants were administered a battery of traditional tests from commonly used neuropsychological test batteries that have been used in research studies with Ugandan adolescents (Table 1) [60-62]. The tests were administered on paper and scored by either a Ugandan Master’s-level clinical psychologist (JLG-O) with expertise in neuropsychological assessment or a trained research assistant (AN) supervised by the psychologist. The research assistant received individualized training in the administration and scoring of the traditional battery by our Ugandan psychologist and a postdoctoral clinical psychology fellow with advanced training in clinical neuropsychology (AS). Individualized training included review of test manuals, observation of assessments, practice assessments, and directly supervised assessments (by JLG-O). Only after approval from the Ugandan psychologist was the research assistant allowed to complete assessments without directly observed supervision. To reduce potential bias, only standardized instruction sets were read, and quality assurance methods were conducted throughout the entirety of the study, such that all completed test protocols were reviewed by the US-based postdoctoral fellow (AS). Tests were administered in a private, quiet office space, with test instructions delivered to children in the local language, and sessions took about 120 to 180 minutes to complete.

The measures assessed 6 neurocognitive domains of functioning: processing speed, working memory, learning, memory, motor speed, and executive functioning. Because we anticipated that most participants would be younger than 18 years and closer to 16 years, we decided to use tests for the younger age range so that all participants would take the same tests. The published upper age limits of the tests are as follows: Kaufman Assessment Battery for Children, Second Edition (KABC-II) [57]—18 years, Children's Color Trails Test (CCTT) [59]—17 years, and Wechsler Intelligence Scales for Children, Fourth Edition (WISC-IV) [58]—16 years. All participants received the same test battery regardless of age; 34 were older than 16 years, including 17 older than 17 years, and 11 older than 18 years. To assess potential ceiling effects among participants older than the upper age limit of these tests, scatterplots were generated and inspected (no evidence of ceiling effects for participants older than the stated age cap for each test was observed).

### Data Analysis

Univariate statistics were used to examine participant demographics, technology use, and acceptability. Prior to analyses, sample-based *z*-scores were computed for each test in both batteries; completion-time raw scores for Trail Making 1, 2, and 3, Number Speed, CCTT1, and CCTT2 were reverse-coded so that higher scores indicated better performance. Global *z*-scores were computed by averaging all available *z*-scores for each test in each battery for each participant. To evaluate the convergent validity of *NeuroScreen*, Pearson correlation coefficients (and 95% CI) were computed between the global *z*-scores and each test within each battery. Kendall rank correlation coefficients were also computed for nonnormally distributed tests; however, they did not affect the overall interpretation of the data, and thus, we report only the Pearson correlation coefficients. HIV status served as the grouping variable, and age (in years), sex (male or female), and education (in years) as covariates in adjusted analyses. Known-groups validity was examined with between-HIV group comparisons across each test in each battery, as well as the global *z*-scores, using 2-tailed *t* tests and age-adjusted analyses of covariance. To examine whether prior tablet experience influenced *NeuroScreen* performance, we compared participants who had previously used a tablet with those who had never used a tablet before using *NeuroScreen* across *NeuroScreen* tests and the global *z*-score, with and without adjustment for age and false discovery rate (FDR) correction. Criterion validity was assessed using Cohen κ by comparing the proportion of participants scoring ≤1 SD and ≤2 SD below the global *z*-score of each battery. Between-battery analyses were restricted to participants with complete data on all tests in both batteries (n=95), and missing data were not imputed. To control for multiple comparisons, the FDR [63] was calculated for each battery’s between-group comparisons. Data were analyzed using R 4.4.1 (R Core Team, 2024) [64].

## Results

### Participant Characteristics

One hundred participants were included in the final analysis. Participants’ mean age (*M*_age_) was 16.6 (SD 1.97; minimum=12.24, maximum=20.80) years; 52% (n=52) were male participants; 92% (n=92) were right-handed. Participants’ mean years of education were 8 (SD 2.36) years. PHIV and control participants did not differ significantly on any demographic variables, except for age (*P*=.03), with the PHIV group being almost 1 year older, on average, than the control group (Table 2). Just about two-thirds of participants in each group chose to complete the Luganda version of *NeuroScreen* over the English version. Five participants were excluded from between-battery analyses owing to incomplete data on one or more tests (4 participants were missing one test on the *NeuroScreen* battery, and 1 participant was missing 2 tests on the traditional battery; these were due to errors in administration). Excluded and included participants did not differ in age, sex, education, or HIV status. For between-battery comparisons, a final total sample of 95 was used, which represented complete data on all tests for both batteries. The mean completion time for the *NeuroScreen* battery was 24.69 (SD 2.80) minutes, with the shortest completion time logged as 20 minutes and the longest as 39 minutes.

**Table 2.** Participant characteristics.

Variable	Full sample (n=100)	PHIV^a^ (n=50)	Controls (n=50)	*t* test or chi-square test (*df*)	*P* value	*d*	
Sex (male), n/N (%)	52/100 (52)	27/50 (54)	25/50 (50)	0.04 (1)^f^	.84	—^b^	
Age (y), mean (SD)	16.16 (1.97)	16.60 (2.04)	15.72 (1.81)	2.27 (98)^g^	.03	−0.46	
Education (y), mean (SD)	8.05 (2.35)	7.86 (2.70)	8.24 (1.95)	−0.81 (98)^g^	.42	0.16	
Monthly income (UGX^c^), mean (SD)	332,597 (311,843)	273,415 (254,181)	400,000 (358,536)	−1.80 (75)^g^	.08	0.41	
Living in extreme poverty^d^, n/N (%)	34/75 (45)	20/41 (49)	14/36 (39)	0.76 (1)^f^	.38	—	
Ever used tablet before^e^, n/N (%)	42/97 (43)	17/50 (34)	25/47 (53)	3.63 (1)^f^	.06	—	
Own a tablet, n/N (%)	4/97 (4)	1/50 (2)	3/47 (6)	0.33 (1)^f^	.57	—	
Frequency of tablet use, mean (SD)	2.12 (1.45)	2.23 (1.57)	2.00 (1.38)	−0.64 (40)^g^	.53	−0.20	
Ease of using tablet^e^, mean (SD)	1.77 (0.79)	1.82 (0.85)	1.72 (0.72)	0.64 (94)^g^	.53	−0.13	
*NeuroScreen* language (Luganda), n (%)	66/100 (66)	32/50 (64)	34/50 (68)	0.04 (1)^f^	.83	—	

a Among participants with perinatally acquired HIV (PHIV), the mean age at ART initiation was 4.79 (SD 3.09) years, the mean nadir CD4 count was 713 (SD 520) cells/mm³, the mean most recent CD4 count was 1048 (SD 452) cells/mm³, and 44/47 (94%) had a most recent viral load of <200 copies/mL.

b Not applicable.

c 1 US$=3781 UGX at the time of writing.

d Extreme poverty defined as <2 US$ per day.

e Three participants were missing data on technology use.

f Chi-square test.

g *t* test.

### Technology Use

Table 2 describes the sample’s experience using tablet devices. Less than half of the sample (42/97, 43%) had ever used a tablet before using the *NeuroScreen* tablet on the day of their study visit. More controls (25/47, 53%) had used tablets than adolescents with PHIV (17/50, 34%), but this was not statistically significant. Of the 42 participants who reported having used a tablet before, 4 (4%) reported currently owning a tablet. Despite limited tablet use and ownership, the mean ease of use rating for the *NeuroScreen* tablet was in the “very easy” to “easy” range (mean 1.79, SD 0.79). There were no differences between groups on this rating. Just under half the sample (48/97, 49%) had ever used a desktop or laptop computer before, and 91% (88/97) reported having used a smartphone with a touchscreen before. There were no between-group differences in either computer or smartphone use. In an unadjusted analysis examining test performance between those who had ever used a tablet and never used a tablet, participants who had used a tablet before performed significantly better on the visual discrimination 1 and visual discrimination 2 tests (*t*_91_=−2.36; *P=*.02 and *t*_95_*=*−2.09; *P=*.04, respectively). However, after adjusting for age and FDR, no differences remained significant.

### Convergent Validity

Pearson correlation coefficients were computed between the global *z*-scores and individual tests in each battery (Multimedia Appendix 1). The correlation between the 2 global *z*-scores was large and statistically significant (*r*=0.75; *P*<.001; 95% CI 0.64-0.83). Correlations between individual tests were generally in the small-to-moderate range, supporting convergent validity between *NeuroScreen* and the traditional battery.

For tests assessing processing speed, correlation coefficients ranged from 0.23 to 0.49. For example, *NeuroScreen* Trail Making 1 was moderately associated with the CCTT1 from the traditional battery (*r*=0.47; *P*<.001; 95% CI 0.29-0.61). Similarly, coding and symbol search from the traditional battery showed moderate correlations with *NeuroScreen* visual discrimination 1 (*r*=0.33; *P=*.001; 95% CI 0.14-0.5; and *r*=0.44; *P*=.001; 95% CI 0.26-0.59, respectively), visual discrimination 2 (*r*=0.40; *P<*.001; 95% CI 0.22-0.55 and *r*=0.49; *P*<.001; 95% CI 0.32-0.62, respectively), and number speed (*r*=0.33; *P=*.001; 95% CI 0.14-0.49 and *r*=0.44; *P*<.001; 95% CI 0.26-0.58, respectively).

For working memory, *NeuroScreen* number span forward was moderately correlated with digit span forward (*r*=0.34; *P*=.001; 95% CI 0.15-0.5), as was *NeuroScreen* number span backward with digit span backward (*r*=0.41; *P*<.001; 95% CI 0.23-0.56). For learning and memory, *NeuroScreen* verbal learning was modestly correlated with the Atlantis paired-associate learning (*r*=0.32; *P*=.001; 95% CI 0.13-0.49), whereas the correlation between delayed memory measures was weaker, with *NeuroScreen* verbal delayed recall showing only a small, nonsignificant association with Atlantis delayed recall (*r*=0.13; *P*=.21; 95% CI −0.07 to 0.31). Verbal learning was moderately correlated with hand movements (*r*=0.36; *P*<.001; 95% CI 0.18-0.52). For executive function tests, *NeuroScreen* Trail Making 2 and CCTT2 were moderately correlated (*r*=0.37; *P*<.001; 95% CI 0.19-0.53). None of the other traditional battery executive function tests correlated with the *NeuroScreen* Trail Making 2 test. The *NeuroScreen* battery did not contain any problem-solving or reasoning tests equivalent to the KABC-II Rover and pattern reasoning subtests; those 2 tests had mostly small correlations with the *NeuroScreen* processing speed and working memory tests. The traditional battery also did not have a pure motor speed test like *NeuroScreen*’s finger tapping, which had small correlations with traditional tests of processing speed, working memory, and learning.

### Known-Groups Validity

We examined whether the *NeuroScreen* and traditional tests, and their global *z*-scores, detected performance differences between groups by HIV status. Across both batteries, participants with PHIV showed poorer performance than controls, although not all test-level differences were statistically significant (Multimedia Appendix 2). This pattern was also evident in the global scores: on the traditional battery, participants with PHIV had a significantly lower mean global *z*-score than controls (mean −0.17, SD 0.62 vs mean 0.17, SD 0.51; *P*=.003; *M*_diff_ =−0.34; 95% CI −0.57 to −0.12). *NeuroScreen* also showed the same significantly lower mean global *z*-score (mean −0.15, SD 0.60 vs mean 0.12, SD 0.52; *P*=.02; *M*_diff_=−0.27; 95% CI −0.50 to −0.04).

Domain-level findings were broadly consistent across the traditional and *NeuroScreen* batteries (Multimedia Appendix 2). Processing speed showed the strongest cross-battery differentiation, with participants with PHIV performing worse than controls on 4 of 8 measures overall (*P*=.001 to *P*=.02). In working memory, both batteries showed the same general pattern of lower performance among participants with PHIV, although no between-group differences were statistically significant. For learning and memory, *NeuroScreen* and traditional measures yielded parallel results, with poorer PHIV performance in unadjusted analyses (learning *P*=.04 to *P*=.047; memory *P*=.04), although these differences were attenuated after adjustment. In motor speed, neither battery differentiated the groups (all *P*≥.34). In executive functioning, both batteries detected poorer PHIV performance on the trail-making switching-type tests in unadjusted analyses, although only the traditional battery difference was statistically significant (*P*=.07 and *P*=.01, respectively). After adjustments, the between-group difference on the traditional test was attenuated.

### Criterion Validity

We examined classification agreement between batteries and HIV groups for participants with global *z*-scores ≤−1 SD and ≤−2 SD below the mean. Using the traditional battery, 15 of 95 participants were classified as ≤−1 SD, compared to 11 of 95 on *NeuroScreen*. Across both batteries, more participants living with PHIV than HIV-negative controls were classified as having global *z*-scores ≤−1 SD. The traditional battery classified significantly more participants with PHIV as ≤−1 SD (13 PHIV and 2 controls; *χ*^2^_1_=9.86; *P=*.001) than the *NeuroScreen* battery (7 PHIV and 4 controls; *χ*^2^_1_=1.0, *P=*.32). Agreement between batteries was as follows: 8 participants were classified as ≤−1 SD by both batteries, 7 were identified only by the traditional battery, and 3 only by *NeuroScreen*. This yielded a κ coefficient of 0.56 (95% CI 0.31-0.80).

At the global *z*-score ≤−2 SD below the mean level, the traditional battery classified 2 of 95 participants as ≤−2 SD, compared with 4 of 95 on *NeuroScreen*; neither difference was statistically significant. Agreement between batteries was as follows: 2 participants were classified as ≤−2 SD by both batteries; 2 additional participants were classified as ≤−2 SD by *NeuroScreen* only; no participants were classified as ≤−2 SD only by the traditional battery. This yielded a κ coefficient of 0.66 (95% CI 0.22-1.0).

## Discussion

In this study, we adapted *NeuroScreen* for use with English- and Luganda-speaking Ugandan adolescents and examined its construct and criterion validity compared with a widely used traditional test battery. Overall, the findings support the use of *NeuroScreen* as a brief, tablet-based approach for assessing neurocognitive functioning in this population. Global performance on *NeuroScreen* was strongly correlated with global performance on the traditional battery, and adolescents with PHIV performed more poorly than HIV-negative controls on both batteries. Tests assessing similar domains had small to moderate correlations.

Convergent validity was most clearly supported by the strong, statistically significant correlation between the global *z*-scores derived from *NeuroScreen* and the traditional battery (*r*=0.75). Correlation coefficients between conceptually similar processing speed tests were among the highest (*r*=0.37-0.48), with conceptually similar tests of attention and working memory having similar correlations (*r*=0.30-0.47), and a conceptually similar executive functioning test showing a moderate correlation (*r*=0.34). These findings are encouraging because an exact one-to-one correspondence between computerized and traditional tests is not necessarily expected, even when they are intended to assess similar domains [65,66]. Differences in modality, response demands, timing parameters, and stimulus format may account for the modest, rather than large, test-level correlations [65-67].

Furthermore, the modest test-level correlations are likely a function of the *NeuroScreen* tests being considerably shorter than the traditional battery tests (eg, coding has a time limit of 120 s vs 45 s for the *NeuroScreen* visual discrimination 2 test, and thus the latter yields fewer scorable responses). Hence, each *NeuroScreen* test comprises fewer items than its traditional counterpart and therefore, by classical test theory, carries less measurement information and lower reliability, meaning briefer tests will correlate more weakly with longer traditional tests even when both assess closely related constructs [68-70]. This is the pattern we observed, and for this reason, the global scores, which aggregate scores across many subtests and thus represent greater length and reliability, are a more appropriate index of convergent validity. This also carries an important implication for *NeuroScreen*. The approximately 25-minute *NeuroScreen* battery shares approximately 56% of the variance with the traditional battery (*r=*0.75 or *r*^2^*=*0.56), which requires 120 to 180 minutes to administer (and more time to score and interpret). *NeuroScreen* captures much of the same signal as the traditional battery at a small fraction of the assessment burden—an efficiency especially valuable in low-resource settings.

*NeuroScreen* was able to distinguish performance between adolescents with PHIV and controls. Adolescents with PHIV had significantly worse global performance on *NeuroScreen* compared with controls. This finding was the same for the traditional battery. Adolescents with PHIV performed worse than controls on all but 2 individual tests and significantly worse on 4 tests in the *NeuroScreen* battery, compared with 6 tests in the traditional battery, and this pattern mostly remained after age adjustment. When adjusting for the FDR on unadjusted comparisons, only 3 of the traditional battery tests remained significant. When adjusting for the FDR on age-adjusted comparisons, only 2 of the traditional battery tests remained significant. Notably, the magnitude of the group differences was similar across batteries, suggesting that *NeuroScreen* was sensitive to the neurocognitive difficulties associated with PHIV in this sample. These findings are consistent with prior work from Uganda and other settings showing that youth with PHIV remain at risk for neurocognitive difficulties despite access to ART [61,62,71]. They also extend prior *NeuroScreen* validation work in PHIV-affected youth and Ugandan clinical populations by demonstrating similar performance with a Luganda-adapted version among Ugandan adolescents [39,49,51].

At the domain level, the most consistent differences between participants with PHIV and controls were observed in processing speed and executive functioning. This pattern is broadly in line with the literature on HIV-associated neurocognitive difficulties in youth, for whom slowed processing speed and executive inefficiencies are among the most commonly reported deficits [9,71,72]. In contrast, differences in learning and memory were significant in unadjusted analyses but were attenuated after age adjustment. Working memory differences were in the expected direction but did not reach statistical significance. These findings suggest that *NeuroScreen* may be especially useful for detecting the domains most consistently affected in PHIV, while also highlighting that not all cognitive domains are equally discriminating in a relatively small sample of virally suppressed youth. Importantly, the absolute differences in mean global *z*-scores between groups were modest, which may reflect the relatively good clinical status of this cohort, all of whom were virally suppressed. Even so, both batteries detected meaningful between-group differences, indicating that subtle but measurable neurocognitive vulnerability persists in this population.

The classification results provide additional support for criterion validity. Both batteries identified more participants with PHIV than controls at ≤1 SD and ≤2 SD below the sample mean global *z*-scores. Between-group differences in classification rates were only statistically significant in the traditional battery at ≤1 SD below the mean threshold. There was a moderate level of agreement between the batteries in their classifications. Given the modest sample size and the small number of participants falling below the classification thresholds, these analyses should be interpreted cautiously. Still, the convergence between the 2 batteries suggests that *NeuroScreen* may have utility not only for continuous assessment of neurocognitive performance but also for identifying youth who may warrant more comprehensive follow-up evaluation.

A major strength of *NeuroScreen* in this context is its feasibility for scale-up. Neurocognitive assessment in many low-resource settings is constrained by shortages of trained specialists, limited availability of culturally and linguistically appropriate tools, and the time burden of traditional batteries. *NeuroScreen* addresses several of these barriers by standardizing administration, automating scoring, and reducing administration time. In its current form, *NeuroScreen* only requires approximately 25 minutes to complete and provides information in line with traditional neuropsychological test batteries, which require considerably more time to complete and score (some batteries can require 2‐6 h just for administration). This is particularly relevant in Uganda and similar LMIC settings, where specialist neuropsychology resources are scarce. The successful Luganda adaptation also underscores the importance of culturally and linguistically grounded test development. Rather than simply translating instructions literally, the adaptation process aimed to preserve meaning and comprehensibility for local users, which is essential for valid assessment [55].

Acceptability findings further support the promise of *NeuroScreen* for use in Uganda. Although many participants had never previously used a tablet, most reported that the app was easy to use. This is notable because familiarity with digital devices is often raised as a concern when introducing tablet-based assessments in low-resource settings [67,73,74]. In this sample, limited prior tablet experience did not appear to prevent successful completion of, or favorable impressions of, the assessment. These findings suggest that, with appropriate orientation and localized instructions, *NeuroScreen* can be acceptable even among youth with limited exposure to this specific technology platform.

Several limitations should be acknowledged. First, this was a relatively small cross-sectional study, which limited statistical power, particularly for impairment classification analyses and finer-grained domain comparisons. Second, the traditional battery used as the comparator is not a true gold standard for neurocognitive assessment in this context. As in many LMIC settings, available traditional neuropsychological tests themselves have limitations related to cultural fit, language, and normative data. Accordingly, this study should be understood as evaluating *NeuroScreen* against a commonly used battery of tests rather than against a definitive reference standard. Relatedly, because no published normative data exist for adolescent populations in Uganda for either battery’s tests, sample-based *z-*scores were used, which can inflate agreement. Third, because all participants with PHIV were virally suppressed and were recruited from an existing urban research cohort, findings may not generalize to youth with less stable HIV disease, those not engaged in care, or more socioeconomically diverse populations. Fourth, several of the traditional measures were administered to participants who exceeded the tests’ published upper age limits (KABC-II, 18 y; CCTT, 17 y; WISC-IV, 16 y). Because we anticipated that most participants would be near 16 years of age and wanted every participant to complete an identical battery, we selected the younger-age forms for the full sample. Administering tests beyond their normed age ranges can affect the validity of age-referenced interpretation and may compress scores near the top of the range, potentially attenuating between-group differences. To assess this, we inspected scatterplots of raw scores against age for each affected test and found no evidence of ceiling effects among older participants. Because our analyses relied on sample-based *z*-scores rather than the tests’ published norms, the influence of exceeding the normative age ranges on our findings is likely limited, though it cannot be fully excluded. Fifth, to optimize study workflow, we did not counterbalance the order of battery administration. As such, order, practice, or fatigue effects on the second (traditional) battery cannot be ruled out. Sixth, we note that the data were collected 4 to 5 years ago. Nonetheless, we do not believe this would have much bearing on interpretation of the findings because the study evaluates psychometric properties of the adapted tests, which are not time-sensitive. In addition, the versions of the *NeuroScreen* and the traditional battery tests have not changed. Furthermore, the clinical profile of the virally suppressed Ugandan adolescents with PHIV has remained largely unchanged. Moreover, the shortage of neuropsychology specialists and valid neuropsychological tests for Luganda-speaking populations also remains. Finally, because this study focused on validity and acceptability, additional work is still needed to establish clinical cut-points, longitudinal sensitivity, and predictive utility for functional outcomes such as school performance, medication adherence, and daily functioning.

Despite these limitations, the present findings have important implications. *NeuroScreen* offers a brief, scalable, and locally adapted approach to neurocognitive assessment that showed a similar pattern of group differences to a traditional neuropsychological test battery. In settings where the use of traditional neuropsychological test batteries is not feasible or practical, a tool such as *NeuroScreen* could expand access to screening and help identify youth who may benefit from monitoring, referral, or intervention. Future studies should examine its performance in larger and more diverse samples, assess test-retest reliability and sensitivity to change over time, and determine how best to integrate *NeuroScreen* into routine HIV care for adolescents in SSA.

## Supplementary material

10.2196/103288 Multimedia Appendix 1 Pearson correlation matrix between *NeuroScreen* and traditional battery tests.

10.2196/103288 Multimedia Appendix 2 Mean test performance across all tests by HIV status.

10.2196/103288 Checklist 1 STROBE checklist.

## Abbreviations

ART: antiretroviral therapy
CCTT: Children's Color Trails Test
FDR: false discovery rate
IRB: institutional review board
JCRC: Joint Clinical Research Centre
KABC-II: Kaufman Assessment Battery for Children, Second Edition
LMIC: low- and middle-income country
NCI: neurocognitive impairment
PHIV: perinatally acquired HIV
SSA: sub-Saharan Africa
STROBE: Strengthening the Reporting of Observational Studies in Epidemiology
WISC-IV: Wechsler Intelligence Scales for Children, Fourth Edition
