# Supplementary material for: Tablet-Based Neurocognitive Tests for Ugandan Adolescents Living With HIV: Cross-Sectional Validation Study
Source: JMIR Mhealth Uhealth. 2026 Oct 13;14:e103288. doi: 10.2196/103288 (PMC13660967; doi:10.2196/103288)
Supplement: Supplementary file 1 [file mhealth-v14-e103288-s001.docx]

| **Table 3.** Pearson Correlation Matrix Between NeuroScreen and Traditional Battery Tests | | | | | | | | | | | | | |
| --- | --- | --- | --- | --- | --- | --- | --- | --- | --- | --- | --- | --- | --- |
|  | **Global *z*** | **Trail Making 1** | **Trail Making 3** | **Visual Discrim 1** | **Visual Discrim 2** | **Number Speed** | **Number Span Forward** | **Number Span Back** | **Verbal Learning** | **Verbal Delayed Recall** | **Trail Making 2** | **Finger Tapping Dom** | **Finger Tapping NonDom** |
| **Global *z*** | 0.75** | 0.57** | 0.34** | 0.50** | 0.56** | 0.52** | 0.39** | 0.54** | 0.46** | 0.17 | 0.38** | 0.33** | 0.29** |
| **CCTT 1** | 0.48** | 0.47** | 0.31** | 0.23* | 0.22* | 0.27** | 0.24* | 0.37** | 0.30** | 0.14 | 0.13 | 0.28** | 0.20* |
| **Coding** | 0.46** | 0.39** | 0.17 | 0.33** | 0.40** | 0.33** | 0.07 | 0.33** | 0.17 | 0.09 | 0.31** | 0.19 | 0.17 |
| **Symbol Search** | 0.59** | 0.43** | 0.24* | 0.44** | 0.49** | 0.44** | 0.30** | 0.40** | 0.25* | 0.10 | 0.28** | 0.26** | 0.24* |
| **Digit Span Forward** | 0.46** | 0.27** | 0.18 | 0.48** | 0.44** | 0.27** | 0.34** | 0.31** | 0.25* | 0.06 | 0.24* | 0.13 | 0.12 |
| **Digit Span Backward** | 0.60** | 0.36** | 0.28** | 0.45** | 0.49** | 0.35** | 0.38** | 0.41** | 0.43** | 0.16 | 0.40** | 0.21* | 0.20* |
| **Atlantis** | 0.43** | 0.33** | 0.09 | 0.21* | 0.34** | 0.38** | 0.13 | 0.30** | 0.32** | 0.25* | 0.09 | 0.23* | 0.25* |
| **Atlantis Delay** | 0.31** | 0.29** | 0.11 | 0.01 | 0.17 | 0.24* | 0.09 | 0.26* | 0.22* | 0.13 | 0.22* | 0.19 | 0.18 |
| **Hand Movements** | 0.39** | 0.20 | 0.25* | 0.25* | 0.30** | 0.24* | 0.30** | 0.12 | 0.36** | 0.17 | 0.08 | 0.21* | 0.23* |
| **CCTT 2** | 0.49** | 0.40** | 0.24* | 0.29** | 0.35** | 0.35** | 0.28** | 0.27** | 0.33** | 0.17 | 0.37** | 0.17 | 0.07 |
| **Rover** | 0.35** | 0.29** | 0.24* | 0.26* | 0.16 | 0.24* | 0.29** | 0.34** | 0.25* | -0.10 | 0.14 | 0.17 | 0.18 |
| **Pattern Reasoning** | 0.27** | 0.23* | 0.09 | 0.28** | 0.25* | 0.22* | 0.11 | 0.37** | 0.10 | -0.11 | 0.20* | 0.10 | 0.02 |
| *Notes.* Bolded variables are *NeuroScreen* tests. All scores have been adjusted (i.e., reserve-coded, if necessary) such that higher scores indicate better performance across all tests in the correlation matrix.  Visual Disc 1 ≡ Visual Discrimination 1, Visual Disc 2 ≡ Visual Discrimination 2, Verbal LL ≡ Verbal List Learning, Verbal LL Del ≡ Verbal List Learning – Delayed; CCTT 1 ≡ Children’s Color Trails Test 1, CCTT 2 ≡ Children’s Color Trails Test 2  * < .05, ** < .01 | | | | | | | | | | | | | |
