# Supplementary material for: Tablet-Based Neurocognitive Tests for Ugandan Adolescents Living With HIV: Cross-Sectional Validation Study
Source: JMIR Mhealth Uhealth. 2026 Oct 13;14:e103288. doi: 10.2196/103288 (PMC13660967; doi:10.2196/103288)
Supplement: Supplementary file 2 [file mhealth-v14-e103288-s002.docx]

| **Table 4.** Neurocognitive Test Performance by HIV Status, With Unadjusted and Age-Adjusted Group Comparisons | | | | | | | | | | | |
| --- | --- | --- | --- | --- | --- | --- | --- | --- | --- | --- | --- |
|  |  | **Full Sample** | **Controls** | **PHIV**^a^ |  |  |  |  |  |  |  |
|  |  | **(N=100)** | **(n=50)** | **(n=50)** |  |  |  |  |  |  |  |
| **Domain** | **Test** | ***M (SD)***^b^ | ***M (SD)*** | ***M (SD)*** | ***t (df)***^c^ | ***P***^d^ | **Cohen d**^e^ | **P**^f^ | ***Adj. t***^g,c^ | **Adj. P**^g,d^ | **Adj. P**^g,f^ |
| Global | ***NeuroScreen***^h^ | -0.01 (0.58) | 0.12 (0.52) | -0.15 (0.60) | 2.33 (94) | .022 | 0.48 | .022 | 2.22 | .029 | .029 |
|  | Traditional | 0.00 (0.59) | 0.17 (0.51) | -0.17 (0.62) | 3.04 (97) | .003 | 0.61 | .006 | 2.36 | .020 | .029 |
| Processing Speed | ***Trail Making Test 1***^i^ | 35.60 (21.01) | 30.88 (17.08) | 40.33 (23.55) | 2.30 (98) | .024 | 0.46 | .17 | 1.99 | .050 | .29 |
|  | ***Trail Making Test 3*** | 20.61 (14.69) | 19.66 (10.90) | 21.57 (17.76) | 0.65 (98) | .52 | 0.13 | .62 | 0.81 | .42 | .50 |
|  | ***Visual Discrimination 1***^j^ | 9.22 (4.31) | 10.00 (4.72) | 8.44 (3.74) | 1.80 (94) | .075 | 0.37 | .18 | 1.66 | .10 | .29 |
|  | ***Visual Discrimination 2*** | 21.71 (5.29) | 22.53 (4.86) | 20.89 (5.62) | 1.56 (98) | .12 | 0.31 | .21 | 1.18 | .24 | .36 |
|  | ***Number Speed*** | 44.90 (13.32) | 43.16 (11.69) | 46.64 (14.67) | 1.31 (98) | .19 | 0.26 | .26 | 1.29 | .20 | .34 |
|  | Children's Color Trails Test 1^k^ | 38.40 (20.80) | 33.44 (11.81) | 43.47 (26.25) | 2.46 (97) | .016 | 0.49 | .043 | 1.89 | .061 | .17 |
|  | WISC-IV: Coding^l^ | 34.15 (10.21) | 37.42 (10.09) | 30.88 (9.32) | 3.37 (98) | .001 | 0.67 | .006 | 2.41 | .018 | .091 |
|  | WISC-IV: Symbol Search | 15.59 (6.53) | 17.90 (5.95) | 13.28 (6.31) | 3.77 (98) | <.001 | 0.75 | .003 | 3.28 | .001 | .016 |
| Working Memory | ***Number Span (Forwards)*** | 3.38 (1.03) | 3.55 (0.90) | 3.20 (1.12) | 1.72 (98) | .088 | 0.35 | .18 | 1.72 | .088 | .29 |
|  | ***Number Span (Backwards)*** | 2.05 (1.00) | 2.19 (0.95) | 1.91 (1.04) | 1.40 (98) | .16 | 0.28 | .25 | 0.83 | .41 | .50 |
|  | WISC-IV: Digit Span Forwards | 7.08 (1.98) | 7.42 (2.12) | 6.74 (1.79) | 1.73 (98) | .086 | 0.35 | .13 | 1.21 | .23 | .34 |
|  | WISC-IV: Digit Span Backwards | 5.66 (1.55) | 5.92 (1.59) | 5.40 (1.47) | 1.70 (98) | .093 | 0.34 | .13 | 0.82 | .41 | .46 |
| Learning | ***Verbal List Learning Total*** | 8.63 (1.22) | 8.88 (1.12) | 8.38 (1.28) | 2.08 (98) | .040 | 0.42 | .17 | 1.68 | .096 | .29 |
|  | KABC-II: Atlantis^m^ | 74.45 (20.35) | 78.48 (18.47) | 70.42 (21.51) | 2.01 (98) | .047 | 0.40 | .086 | 1.15 | .26 | .34 |
|  | KABC-II: Hand Movements | 8.12 (3.15) | 8.42 (3.12) | 7.82 (3.19) | 0.95 (98) | .34 | 0.19 | .42 | 1.08 | .28 | .34 |
| Memory | ***Verbal List Learning Delayed*** | 3.78 (1.19) | 4.02 (0.94) | 3.54 (1.37) | 2.04 (98) | .044 | 0.41 | .17 | 1.29 | .20 | .34 |
|  | KABC-II: Atlantis Delayed | 12.90 (5.76) | 14.06 (5.81) | 11.74 (5.53) | 2.05 (98) | .044 | 0.41 | .086 | 1.25 | .21 | .34 |
| Executive Functioning | ***Trail Making Test 2*** | 49.30 (25.89) | 44.63 (24.91) | 53.96 (26.26) | 1.82 (98) | .071 | 0.37 | .18 | 1.56 | .12 | .29 |
|  | Children's Color Trails Test 2^k^ | 72.36 (35.66) | 63.48 (28.67) | 81.43 (39.89) | 2.57 (97) | .012 | 0.52 | .042 | 2.28 | .025 | .091 |
| Problem Solving | KABC-II: Pattern Reasoning | 15.34 (9.60) | 14.84 (9.45) | 15.84 (9.82) | 0.52 (98) | .60 | −0.10 | .64 | 1.17 | .25 | .34 |
|  | KABC-II: Rover | 22.21 (9.15) | 21.78 (9.30) | 22.64 (9.06) | 0.47 (98) | .64 | −0.09 | .64 | 0.06 | .95 | .95 |
| Motor Speed | ***Finger Tapping (Dominant)*** | 210.50 (52.27) | 208.04 (49.64) | 212.96 (55.18) | 0.47 (98) | .64 | −0.09 | .70 | 0.42 | .67 | .73 |
|  | ***Finger Tapping (NonDominant)*** | 196.52 (44.67) | 195.00 (44.14) | 198.04 (45.58) | 0.34 (98) | .74 | −0.07 | .74 | 0.06 | .95 | .95 |
|  | | | | | | | | | | | |

*Note. Per-test degrees of freedom vary because of item-level missing data.*

^a^ PHIV: adolescents living with perinatally acquired HIV.

^b^ M: mean; SD: standard deviation. Global scores are standardized (z) composites with a full-sample mean of 0.

^c^ t (df): independent-samples t test statistic and degrees of freedom; absolute values are shown, with the direction of the difference indicated by the group means and by Cohen d.

^d^ P: unadjusted 2-tailed P value; P<.001 is shown where the value rounds below .001.

^e^ Cohen d: standardized mean difference; positive values indicate better performance in the control group and negative values indicate nominally better performance in the PHIV group.

^f^ P (FDR): Benjamini-Hochberg false discovery rate–adjusted P value.

^g^ Adj.: adjusted for age.

^h^ Tests shown in bold italics belong to the NeuroScreen battery; all other tests belong to the traditional battery.

^i^ Timed tests (Trail Making Test, Children's Color Trails Test, Number Speed) are reported as completion times in seconds, for which higher values indicate poorer performance; for all other tests higher scores indicate better performance.

^j^ Four participants were missing this measure (n=96).

^k^ One participant was missing this measure (n=99).

^l^ WISC-IV: Wechsler Intelligence Scale for Children, 4th edition.

^m^ KABC-II: Kaufman Assessment Battery for Children, 2nd edition.
