# Supplementary material for: Tablet-Based Neurocognitive Tests for Ugandan Adolescents Living With HIV: Cross-Sectional Validation Study
Source: JMIR Mhealth Uhealth. 2026 Oct 13;14:e103288. doi: 10.2196/103288 (PMC13660967; doi:10.2196/103288)
Supplement: Supplementary file 3 [file mhealth-v14-e103288-s003.docx]

**STROBE Statement**

| **Item** **No.** | **Recommendation** | **Reported on page(s)** |
| --- | --- | --- |
| **Title and Abstract** | | |
| **1** | (a) Indicate the study’s design with a commonly used term in the title or the abstract. | 1 |
|  | (b) Provide in the abstract an informative and balanced summary of what was done and what was found. | 2–3 |
| **Introduction** | | |
| **2** | Background/rationale: Explain the scientific background and rationale for the investigation being reported. | 4–5 |
| **3** | Objectives: State specific objectives, including any prespecified hypotheses. | 5 |
| **Methods** | | |
| **4** | Study design: Present key elements of study design early in the paper. | 6 |
| **5** | Setting: Describe the setting, locations, and relevant dates, including periods of recruitment, exposure, follow-up, and data collection. | 7 |
| **6** | (a) Participants: Give the eligibility criteria, and the sources and methods of selection of participants. | 6–7 |
|  | (b) (For matched studies) Give matching criteria. | Not applicable (unmatched design) |
| **7** | Variables: Clearly define all outcomes, exposures, predictors, potential confounders, and effect modifiers. Give diagnostic criteria, if applicable. | 11 |
| **8** | Data sources/measurement: For each variable of interest, give sources of data and details of methods of assessment. Describe comparability of assessment methods if there is more than one group. | 8–10 |
| **9** | Bias: Describe any efforts to address potential sources of bias. | 9–10; 19 |
| **10** | Study size: Explain how the study size was arrived at. | 7 |
| **11** | Quantitative variables: Explain how quantitative variables were handled in the analyses. If applicable, describe which groupings were chosen and why. | 11 |
| **12** | (a) Statistical methods: Describe all statistical methods, including those used to control for confounding. | 11 |
|  | (b) Describe any methods used to examine subgroups and interactions. | 11 |
|  | (c) Explain how missing data were addressed. | 11 |
|  | (d) (Cross-sectional) If applicable, describe analytical methods taking account of sampling strategy. | Not applicable (convenience sample; no weighting) |
|  | (e) Describe any sensitivity analyses. | 11; 13–15 |
| **Results** | | |
| **13** | (a) Participants: Report the numbers of individuals at each stage of the study. | 11–12 |
|  | (b) Give reasons for non-participation at each stage. | 11–12 |
|  | (c) Consider use of a flow diagram. | Not reported |
| **14** | (a) Descriptive data: Give characteristics of study participants and information on exposures and potential confounders. | 12–13 |
|  | (b) Indicate the number of participants with missing data for each variable of interest. | 12–13 (Table 2) |
|  | (c) (Cohort) Summarize follow-up time. | Not applicable (single time point) |
| **15** | Outcome data (Cross-sectional): Report numbers of outcome events or summary measures. | 13–15 |
| **16** | (a) Main results: Give unadjusted estimates and, if applicable, confounder-adjusted estimates and their precision (e.g., 95% CI). Make clear which confounders were adjusted for and why they were included. | 13–15 |
|  | (b) Report category boundaries when continuous variables were categorized. | 15 |
|  | (c) If relevant, consider translating estimates of relative risk into absolute risk for a meaningful time period. | Not applicable |
| **17** | Other analyses: Report other analyses done (e.g., analyses of subgroups and interactions, and sensitivity analyses). | 12 |
| **Discussion** | | |
| **18** | Key results: Summarize key results with reference to study objectives. | 15–16 |
| **19** | Limitations: Discuss limitations of the study, taking into account sources of potential bias or imprecision. Discuss both direction and magnitude of any potential bias. | 18–19 |
| **20** | Interpretation: Give a cautious overall interpretation of results considering objectives, limitations, multiplicity of analyses, results from similar studies, and other relevant evidence. | 16–20 |
| **21** | Generalizability: Discuss the generalizability (external validity) of the study results. | 19 |
| **Other Information** | | |
| **22** | Funding: Give the source of funding and the role of the funders for the present study and, if applicable, for the original study on which the present article is based. | 20 |
